# Transcriptomic analysis reveals insights into deep-sea adaptations of the dominant species, *Shinkaia crosnieri* (Crustacea: Decapoda: Anomura), inhabiting both hydrothermal vents and cold seeps

**DOI:** 10.1186/s12864-019-5753-7

**Published:** 2019-05-18

**Authors:** Jiao Cheng, Min Hui, Zhongli Sha

**Affiliations:** 1 Laboratory of Marine Organism Taxonomy and Phylogeny, Institute of Oceanology, Chinese Academy of Sciences, Qingdao, 266071 China; 2 Laboratory for Marine Biology and Biotechnology, Qingdao National Laboratory for Marine Science and Technology, Qingdao, 266237 China; 3 Center for Ocean Mega-Science, Chinese Academy of Sciences, Qingdao, 266071 China; 4 University of Chinese Academy of Sciences, Beijing, 100049 China

**Keywords:** Galatheidae, Deep-sea adaptation, Comparative transcriptome, Positive selection, Differential expression

## Abstract

**Background:**

Hydrothermal vents and cold seeps are typical deep-sea chemosynthetically-driven ecosystems that allow high abundance of specialized macro-benthos. To gather knowledge about the genetic basis of adaptation to these extreme environments, species shared between different habitats, especially for the dominant species, are of particular interest. The galatheid squat lobster, *Shinkaia crosnieri* Baba and Williams, 1998, is one of the few dominant species inhabiting both deep-sea hydrothermal vents and cold seeps. In this study, we performed transcriptome analyses of *S. crosnieri* collected from the Iheya North hydrothermal vent (HV) and a cold seep in the South China Sea (CS) to provide insights into how this species has evolved to thrive in different deep-sea chemosynthetic ecosystems.

**Results:**

We analyzed 5347 orthologs between HV and CS to identify genes under positive selection through the maximum likelihood approach. A total of 82 genes were identified to be positively selected and covered diverse functional categories, potentially indicating their importance for *S. crosnieri* to cope with environmental heterogeneity between deep-sea vents and seeps. Among 39,806 annotated unigenes, a large number of differentially expressed genes (DEGs) were identified between HV and CS, including 339 and 206 genes significantly up-regulated in HV and CS, respectively. Most of the DEGs associated with stress response and immunity were up-regulated in HV, possibly allowing *S. crosnieri* to increase its capability to manage more environmental stresses in the hydrothermal vents.

**Conclusions:**

We provide the first comprehensive transcriptomic resource for the deep-sea squat lobster, *S. crosnieri*, inhabiting both hydrothermal vents and cold seeps. A number of stress response and immune-related genes were positively selected and/or differentially expressed, potentially indicating their important roles for *S. crosnieri* to thrive in both deep-sea vents and cold seeps. Our results indicated that genetic adaptation of *S. crosnieri* to different deep-sea chemosynthetic environments might be mediated by adaptive evolution of functional genes related to stress response and immunity, and alterations in their gene expression that lead to different stress resistance. However, further work is required to test these proposed hypotheses. All results can constitute important baseline data for further studies towards elucidating the adaptive mechanisms in deep-sea crustaceans.

**Electronic supplementary material:**

The online version of this article (10.1186/s12864-019-5753-7) contains supplementary material, which is available to authorized users.

## Background

The discoveries of chemosynthetic ecosystems (hydrothermal vents, cold seeps and other deep-sea sites of organic enrichment) have revolutionized our perceptions of life in the deep sea [1]. Hydrothermal vents and cold seeps constitute energy hotpots on the seafloor that share high concentrations of reduced chemicals as energy source utilized by chemoautotrophic bacteria, supporting a high-biomass benthic community far from the euphotic zone [2]. Despite sharing the similar process of chemosynthesis, hydrothermal vents seem more severe and ephemeral than cold seeps as environments for organisms [3]. Hydrothermal vents are patchily distributed in mid-ocean ridges, volcanic arcs and back-arc spreading centers, where they are usually ephemeral and not strongly sedimented. In comparison, more stable and sediment-hosted cold seeps occur at both passive continental margins and subduction zones, where more stable communities lead to species with extreme lifespan [4]. Active emitting sites in hydrothermal vents discharge more dissolved organic chemicals, including methane, hydrogen sulfide and other hydrocarbon-rich fluids, than oozing sites in cold seeps [3]. On the other hand, vent effluents are also known to enrich in heavy metals [5], reaching such high concentrations that is considered to be toxic to living organisms [6]. Apart from geological settings and the chemicals used for energy, hydrothermal vents have an appreciable temperature gradient from the maximum of emitting hot water (ca. 350 °C) to the minimum of ambient cold water (2 °C–3 °C), but no such gradient in cold seeps [7].

Despite their habitat differences, deep-sea vent and seep communities share more than 20% species occurring in the same region, indicating their genetic connectivity [8]. Beyond biogeographic issues, another hot topic in deep-sea biology is how those animals have adapted to the extreme environmental conditions including high hydrostatic pressure, variable temperatures and high levels of toxins. Advances in sequencing technologies over the last few years have allowed researchers to uncover molecular processes important for fauna that can survive under such inhospitable environments in the deep sea. Within deep-sea chemosynthetic ecosystems, particularly hydrothermal vents, transcriptome sequencing has been applied in several deep-sea macro-benthos to explore the genetic basis of their adaptation to extreme environmental conditions, including mussels [9, 10], worms [11, 12] and shrimps [13–15]. Among them, a high-throughput sequencing analysis was conducted in the cold seep mussel *Bathymodiolus platifrons*, in which genes related to immune function and detoxification were identified to be responsible for extreme environmental adaptation [15]. In addition, a comparative transcriptome analysis of the alvinocaridid shrimp *Rimicaris* sp. indicated that most of the genes involved in sulfur metabolism and detoxification were up-regulated in the shrimps taken directly from the vent site compared with those maintained under normal laboratory condition [16]. These studies greatly extend our understanding on the molecular mechanisms of deep-sea adaptations. To gain more knowledge about the genetic basis of adaptation to deep-sea chemosynthetic environments, shared species across different kinds of deep-sea chemosynthetic habitats, especially for the dominant species, are of particular interest [17, 18].

The galatheid squat lobster, *Shinkaia crosnieri* Baba and Williams, 1998, is first described from hydrothermally active areas of the Bismarck Archipelago and the Okinawa Trough in the west Pacific Ocean [19]. Chan et al. [20] also mentioned this species as the first known deep-sea hydrothermal animal in Taiwan. Besides inhabiting many deep-sea hydrothermal vents, *S. crosnieri* has also been considered the dominant member of the fauna at a deep-sea cold seep on the Formosa Ridge off the coast of southwestern Taiwan in the South China Sea [21]. Recent molecular studies revealed high levels of genetic differentiation between the vent and seep populations for *S. crosnieri*, but not for the mussel *B. platifrons*, and suggested relatively low degree of tolerance to environmental heterogeneity of *S. crosnieri* in comparison to *B. platifrons* [22]. Therefore, *S. crosnieri* may serve as an ideal model to study how marine organisms have adapted to different deep-sea chemosynthetic environments.

Given the environmental heterogeneity between deep-sea vents and cold seeps, we hypothesized that the species co-distributed in both environments likely have evolved survival strategies to adapt to their respective habitats. In order to reveal the possible differences of molecular basis of *S. crosnieri* in adaptation to hydrothermal vents and cold seeps, we conducted a high-throughput transcriptome analysis of the squat lobsters collected from the Iheya North hydrothermal vent in Okinawa Trough (HV) and a cold seep in the South China Sea (CS). With these datasets, we aim to trace signatures of positive selection in *S. crosnieri* during adaptation to deep-sea vents and seeps, and discover genes and pathways that are differentially expressed between HV and CS. By comparing the sequence characteristics and gene expression between the vent and seep *S. crosnieri*, we also hope to identify genes that potentially contributed to the survival of deep-sea squat lobster in both environments, thus providing insights into how this species has evolved to thrive in different deep-sea chemosynthetic ecosystems. These results can constitute important baseline data with which to gain insights into the adaptation process to deep-sea extreme environments in macro-organisms.

## Methods

### Sample collection and RNA isolation

Specimens of *S. crosnieri* were collected using suction samplers mounted on remotely operated vehicle (ROV) ‘Fa Xian’ from the Iheya North hydrothermal vent in Okinawa Trough (126°58.82′E, 27°47.44′N, ~ 983.5 m) in April 2014, and a cold seep in the South China Sea (119°28.54′E, 22°11.57′N, ~ 1133.4 m) in July 2015 during the cruises conducted by the scientific research vessel KEXUE. Once board, the squat lobsters were immediately frozen in liquid nitrogen (sacrificed) and stored at − 80 °C until RNA extraction. Every effort was made to minimize animal suffering. Three adult individuals from each sampling site were randomly selected as biological replications. Total RNA for the whole bodies of six *S. crosnieri* individuals was extracted separately using the mirVana™ miRNA Isolation Kit without phenol (Ambion, Inc., Austin, TX, USA) and treated with RNase free DNaseI according to manufacturer’s instructions. The quality of RNA samples was detected by 1% agarose gel electrophoresis stained with GelRed (Biotium, Fremont, CA, USA) and further confirmed by spectrophotometer (NanoDrop ND-1000, NanoDrop Technologies, Wilmington, DE, USA).

### Library construction, sequencing and de novo assembly

A total amount of 1.5 μg RNA per sample was used to construct cDNA library. Six libraries were constructed using NEBNext® Ultra™ RNA Library Prep Kit (NEB, USA) following the manufacture’s recommendations. Briefly, poly (A) mRNA was purified from total RNA using oligo (dT)-attached magnetic beads and then broken into short fragments to synthesize double-stranded cDNA using random hexamer primer and M-MuLV reverse transcriptase (Superscript II, Life Technologies, CA, USA). The synthesized cDNA was subjected to end-repair, phosphorylation, 3′ adenylation, and ligation of adapters. Afterwards, cDNA libraries were generated by polymerase chain reaction (PCR), and the library preparations were sequenced on an Illumina HiSeqTM 2500 sequencing platform (commercial service) in pair-end mode with a read length of 100 bp.

To obtain clean reads, raw reads were cleaned by removing adapter sequences, low quality bases (Length threshold value > 35 bp), 3′-end low quality bases (Quality threshold value > 20) and the reads containing ploy-N. The quality of raw data was evaluated using FastQC software (http://www.bioinformatics.babraham.ac.uk/projects/fastqc/), and low quality reads were discarded by using NGS QC Toolkit v2.3.3 software (http://www.nipgr.ac.in/ngsqctoolkit.html). Clean reads were assembled into contigs using Trinity software (version: trinityrnaseq_r20131110) with min_kmer_cov set to 2 and all other parameters set to default [23]. The generated contigs were clustered into transcripts by using TGICL software system [24]. The longest one of each transcript set was defined as a unigene for further functional annotation.

### Functional annotation and protein-coding sequence prediction

All unigenes were searched against public databases, including NCBI Nr (non-redundant protein; E-value *<*1E-5), SwissProt (http://www.ebi.ac.uk/uniprot/; E-value *<*1E-5), KOG (euKaryotic Ortholog Group; http://www.ncbi.nlm.nih.gov/COG/; E-value *<*1E-3), GO (Gene Ontology; http://www.geneontology.org/; E-value <1E-6) and KEGG (Kyoto Encyclopedia of Genes and Genomes; http://www.genome.jp/kegg/; E-value *<*1E-10) databases. Genes encoding protein domains were identified by searching against Pfam (Protein Family; http://pfam.xfam.org/; E-value *<* 0.01) database. GO classification was conducted using Blast2GO v2.5 [25] with an E-value of 1E-6, and KEGG classification was performed using KASS (vr140224) [26] and the KEGG Automatic Annotation Server.

In specific cases, putative functions of *S. crosnieri* protein were further investigated by examining multiple-sequence alignments for conserved functional domains and/or by performing phylogenetic analyses. In those instances, searches against the NCBI Nr database using BLAST were conducted to obtain homologous sequences. Alignment of amino acid sequences was performed using MUSCLE v3.8.31 [27]. The phylogenetic tree was conducted on the basis of amino acid sequence alignment using the maximum likelihood method as implemented in PhyML v3.0 [28]. The node reliability was calculated from 100 bootstrap replications. In addition, functional domain analysis was performed with SMART (http://smart.embl.de/).

### Identification of genes under positive selection

Detection of genes that have undergone positive selection can provide insights into the process of adaptive evolution [29]. In the present study, we performed a maximum likelihood approach to detect signals of positive selection in *S. crosnieri* inhabiting both hydrothermal vents and cold seeps. We followed the method of comparative transcriptomic analyses to identify one-to-one orthologs. Blast-based approach was performed to extract the coding sequences (CDS) of each putative unigene according to the BLASTX results. For sequences that did not have alignment results, the software ESTScan [30] was used to determine their directions. The amino acid sequences were acquired by translating the CDS using the standard codon table. Reciprocal BLASTP was conducted for all amino acid sequences with a cut-off E-value of 1E-5. OrthoMCL v2.0.3 [31] was employed to construct orthologous groups using a Markov Cluster algorithm with default settings. Amino acid sequence alignment and phylogenetic tree construction were both followed the methods mentioned above.

The ratio (ω) of the non-synonymous (d_N_) nucleotide substitution rate to the synonymous (d_S_) nucleotide substitution rate can be used as an indicator of selective pressure operating on a protein-coding gene. The ratio ω > 1 indicates positive selection, ω = 1 suggests neutral selection, and ω < 1 represents negative selection [32]. We employed the site model that allowed ω to vary among sites to assess positive selection episodes affecting specific amino acid sites as implemented in the CODEML program in the PAML 4 package [33]. Likelihood ratio tests (LRTs) were conducted to calculate log-likelihood values between a neutral model that provided two codon site categories with ω < 1 and ω = 1 respectively and an alternative model that provided another category for sites with ω > 1, which indicated positive selection. Two times the difference between the log-likelihood values for the two models were used to perform chi-square test with two degrees of freedom. The *P* values were computed based on the chi-square statistic and further adjusted by the False Discovery Rate (FDR) method. Afterwards, the Bayes Empirical Bayes (BEB) method was applied to calculate the posterior probability (PP) for site classes to identify sites under positive selection [34]. Codon sites with the PP value > 0.95 were treated as positively selected sites. Genes with adjusted *P* values < 0.05 and with positively selected sites were termed as positively selected genes (PSGs) in the following analysis.

### Quantification and functional analysis of differentially expressed genes

Gene expression levels in each sample were estimated by mapping clean reads to the assembled transcriptome to obtain read counts for each gene using RSEM [35]. The abundance of all genes was normalized and calculated using uniquely mapped reads by the expected number of Fragment Per Kilobase of transcript sequence per Millions (FPKM) base pairs sequenced method, which takes into account the influence of both sequencing depth and gene length on read count [36]. Differential expression analysis was implemented using the DESeq R package v1.12.0 based on the negative binomial distribution [37]. The DESeq normalization method was applied to normalize read counts as it is widely studied and generally performs well relative to other methods [38]. After normalization, differential expression hypothesis testing was performed on the data. *P* values were corrected for multiple testing using the Benjamini–Hochberg’s method [39]. In this study, the adjusted *P* value (*P*adj) < 0.05 and the absolute value of the log2 (fold change) > 1 were used as the thresholds to select differentially expressed genes (DEGs) between the vent and seep datasets.

Functional enrichment analyses including GO and KEGG pathway were performed to investigate which GO items and metabolic pathways the DEGs participated in, respectively. Specifically, GO enrichment analysis of candidate genes was implemented in the GOseq R package [40], which can eliminate gene length bias. To circumvent the problem of multi-testing, the FDR approach was applied to correct significant levels [41]. GO terms with an FDR threshold of 0.05 were considered significantly enriched by DEGs. For KEGG enrichment analysis, candidate gene lists were incorporated to the KEGG database to identify genes involved in particular biological processes and pathways that occur at a higher frequency than would be expected for a randomly selected set of genes. Here, we used KOBAS software v2.0.12 [42] to test the statistical enrichment of candidate genes in KEGG pathways. The pathways with FDR less than 0.05 were defined to be statistically represented. In addition, the DEG datasets were hierarchically clustered and visualized by using Pheatmap v1.0.8 of R package [43] to estimate expression patterns of DEGs between HV and CS.

### Quantitative real-time PCR (qRT-PCR) verification

To validate the expression level of the DEGs obtained by RNA-Seq, qRT-PCR was conducted with the RNA used for transcriptome sequencing. The first-strand cDNA was synthesized by using M-MLV reverse transcriptase (Promega, Madison, USA) and oligodT. The SYBR Green RT PCR assay was performed in an ABI PRISM 7300 Sequence Detection System (Applied Biosystems). Ten pairs of gene-specific primers (Additional file 1: Table S1) were used to amplify the partial cDNA sequences, respectively. All experiments were conducted three times. The relative expression level of the target genes was calculated using the 2^−ΔΔCt^ method with the *β-actin* gene as internal control. The results were subjected to one-way analysis of variance (ANOVA) using SPSS 17.0 software (SPSS Inc., Chicago, IL, USA), and the cut-off *P* value of 0.05 was considered statistically significant.

## Results

### De novo assembly and functional annotation of *S. crosnieri* transcriptomes

A total of 430,940,036 raw reads were generated from *S. crosnieri* transcriptomes, of which 222,129,956 raw reads from vent samples and 208,810,080 raw reads from seep samples. All raw-sequence reads data have been deposited in the NCBI Sequence Read Archive (SRA) database under the accession number SRP151635. After filtering, 20.17G and 19.62G clean bases were obtained for the vent and seep samples, respectively. Over 98% clean Illumina reads in all samples exceeded Q20, indicating high quality of the sequencing data. Then clean reads were assembled into 189,543 transcripts with an average length of 583 bp and N50 value of 796 bp, and 108,237 unigenes with an average length of 838 bp and N50 value of 1125 bp. The statistics for the data output and de novo assemblies are summarized in Table 1.

**Table 1:** Summary of assembling and functional annotation of *S. crosnieri* transcriptomes

	Hydrothermal vent	Cold seep	Total
*Sequencing data statistics*			
Raw reads	222,129,956	208,810,080	430,940,036
Clean bases (Gb)	20.17	19.62	39.79
Clean reads	161,376,548	157,076,052	318,452,600
*Assembly statistics*			
Number of transcripts	189,543		
Mean length of transcripts	583		
N50 of transcripts	796		
Number of unigenes	108,237		
Mean length of unigenes	838		
N50 of unigenes	1125		
*Functional annotation*			
NCBI non-redundant (nr) database	36,114 (33.37%)		
SwissProt	32,419 (29.95%)		
KOG	28,605 (26.43%)		
KEGG	13,588 (12.55%)		
GO	30,486 (28.17%)		
Annotated in all database	11,140 (10.29%)		
Annotated in at least one database	39,806 (36.78%)		

A total of 39,806 (36.78%) unigenes had at least one significant hit in the Nr, SwissProt, KOG, GO, or KEGG database (Table 1). Based on Nr annotations, we used the GO classification system to functionally categorize unigenes. In total, 30,486 (28.17%) unigenes were successful annotated by GO analysis and were assigned to three major GO categories: biological process (138,448, 45.86%), cellular component (121,617, 40.29%) and molecular function (41,819, 13.85%) (Additional file 2: Figure S1A). These GO terms were further subdivided into 64 subcategories. In KOG analysis, the main function classifications were found to be ‘General function prediction only’, ‘Signal transduction mechanisms’, ‘Posttranslational modification, protein turnover, chaperones’ and ‘Translation, ribosomal structure and biogenesis’ (Additional file 2: Figure S1B). In addition, 13,588 unigenes were mapped to 350 KEGG pathways, with ‘Signal transduction’ being the most abundant category followed by ‘Carbohydrate metabolism’ and ‘Translation’ (Additional file 2: Figure S1C). These annotation and classification will aid understanding of the gene function in *S. crosnieri*.

### PCGs between HV and CS

A total of 5347 orthologous unigene pairs were identified in *S. crosnieri* between HV and CS and the phylogenic tree was constructed with the other two arthropods *Daphnia pulex* and *Drosophilla virilis* (Additional file 3: Figure S2). Of these orthologous genes, 82 were identified as PCGs with adjusted *P* values smaller than 0.05 and with the BEB posterior probability of sites larger than 0.95 (Additional file 4: Table S2). Further analysis indicated that the PCGs between HV and CS included thioredoxin (Trx), glutathione S-transferase (GST), and rhodanese involved in antioxidation and detoxification; C-type lectin (CTL), antimicrobial peptide (AMP), heparan sulfate proteoglycans (HSPG) contributing to immune defense; DNA repair protein, splicing factor, transcription factor, transcriptional adapter 2B, p53 protein, ATP-dependent DNA helicase, zinc finger protein participating in genetic information processing (Table 2). The very abundant PCGs related to various functions between HV and CS are indicative of a strong selective force that *S. crosnieri* might undergo during the adaptation process to different deep-sea chemosynthetic environments.

**Table 2:** PSGs related to stress response, immunity and genetic information processing between vent and seep *S. crosnieri*

Gene category	Gene description	*P*-value	Adjusted *P*-value
*Stress response and immunity*			
*Trx*	Thioredoxin domain-containing protein 11	3.23E-14	4.01E-12
*GST*	Glutathione S-transferase 11	5.51E-10	5.03E-08
*RHOD*	Rhodanese 1-like protein	8.75E-04	2.66E-02
*CTL*	C-type lectin 2	1.39E-04	5.14E-03
*HSPG*	Basement membrane-specific heparan sulfate proteoglycan core protein isoform X15	4.83E-07	2.88E-05
*AMP*	Antimicrobial peptide type 2 precursor Iib	6.72E-05	2.55E-03
*GILT*	Gamma-interferon-inducible lysosomal thiol reductase	6.27E-07	3.54E-05
*Genetic information processing*			
*RAD4*	DNA repair protein RAD4	7.98E-04	2.45E-02
*SRSF*	Serine/arginine-rich splicing factor	1.70E-07	1.12E-05
*BTF3*	RNA polymerase II proteinral transcription factor BTF3	2.00E-15	3.65E-13
*ADA2B*	Transcriptional adapter 2B	0	0
*p53*	p53 protein	1.40E-10	1.32E-08
*DNA2*	ATP-dependent DNA helicase 2	1.51E-04	5.46E-03
*EZH*	Enhancer of zeste protein	1.19E-03	3.56E-02
*ZNF268*	Zinc finger protein 268	3.18E-07	2.01E-05

### DEGs between HV and CS

**Fig. 1:**
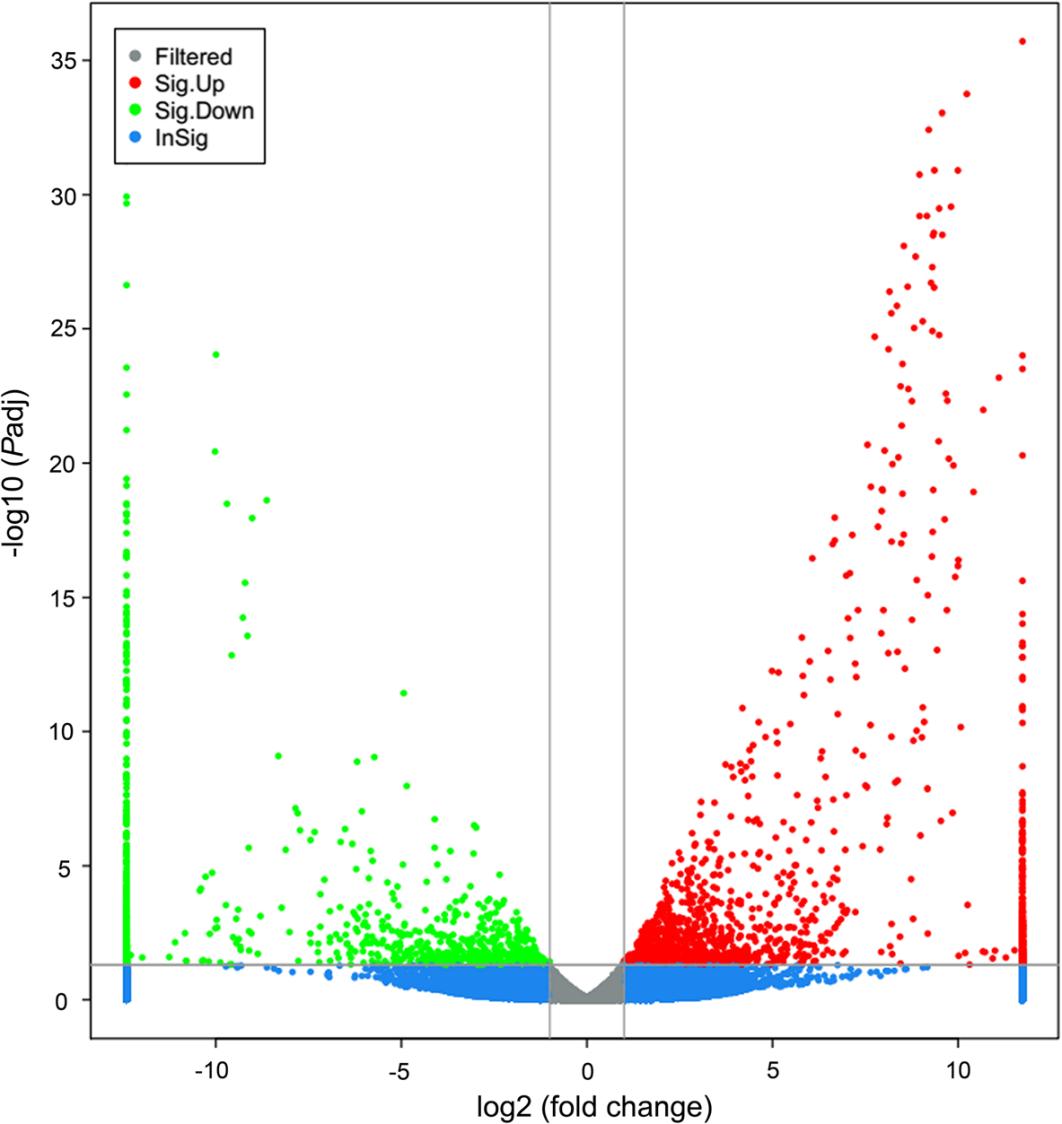
Volcano plot showing DEGs between HV and CS. The red and green dots represent up-regulated and down-regulated DEGs in HV, respectively; the blue dots represent non-DEGs. x-axis: log2 fold change; y-axis: −log10 (*P*adj) for each DEG. vertical dotted lines: fold change ≥ 2 or ≤ 2; horizontal dotted line: the significance cut-off (*P*adj value = 0.05)

**Fig. 2:**
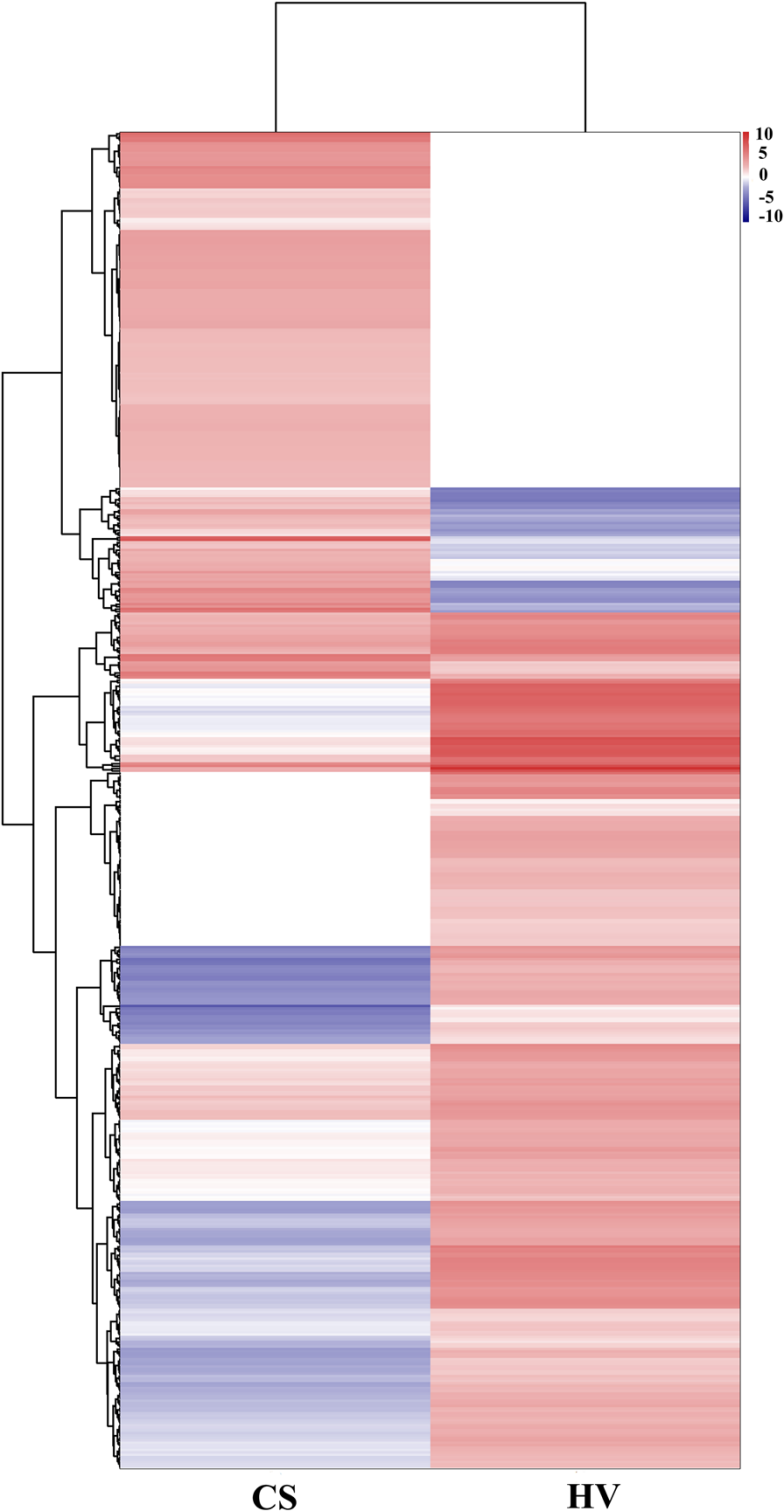
Hierarchical clustering analysis for the 545 DEGs between HV and CS transcriptomes. The clustering indicates similar expression patterns among *S. crosnieri* samples (x-axis) and among the genes (y-axis). The expression level is represented by color intensities (red color indicates the higher expression, and blue color indicates the lower expression of the gene)

**Fig. 3:**
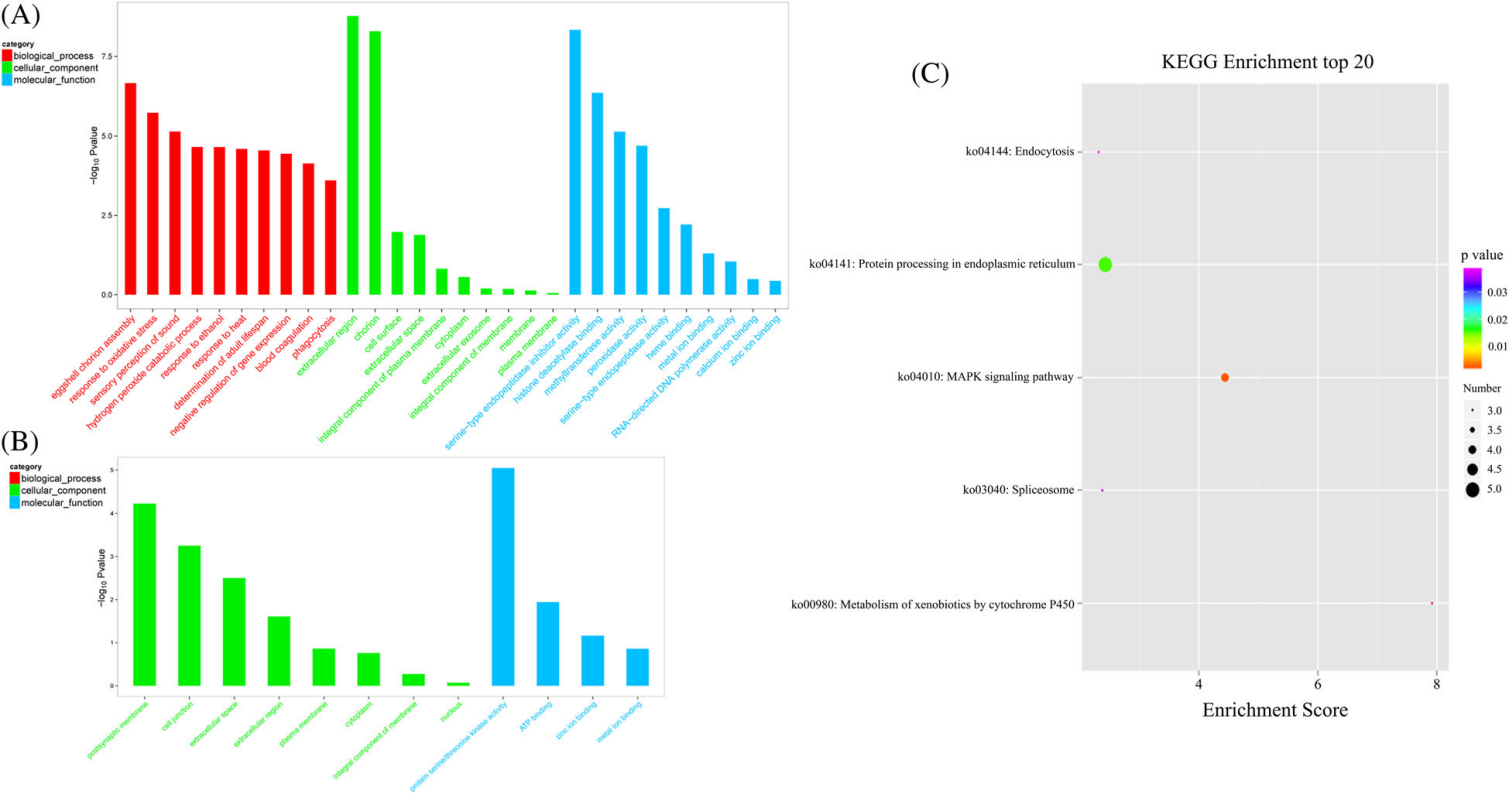
GO enrichment analysis of up-regulated DEGs (**a**) and down-regulated DEGs (**b**) in HV samples with GO IDs that were categorized into three main categories shown in different colors: biological process (red), cellular component (green), and molecular function (blue). KEGG enrichment analysis of up-regulated DEGs in HV (**c**) was also shown

A consistent expression tendency between the results based on qRT-PCR and RNA-Seq data (Additional file 1: Table S1) was detected, showing the accuracy of the RNA-Seq. A total of 545 unigenes were differentially expressed between vent and seep *S. crosnieri* samples. Among them, 339 (62.2%) genes showed higher expression levels in HV than in CS and 206 (37.8%) genes showed lower expression levels in HV than in CS (Additional file 5: Table S3). The relation between the FDR and FC (fold change) for all DEGs is shown in the volcano plots (Fig. 1). The expression heatmap showed remarkably different expression patterns of 545 DEGs (Fig. 2) with detailed information in Additional file 6: Figure S3, which revealed the differences between the adaptation to hydrothermal vents and cold seeps in *S. crosnieri*. Of the 545 DEGs, 193 were annotated to 439 GO terms accompanied by 145 and 48 genes with relatively high expression in HV and CS, respectively. In comparison with CS, more up-regulated subcategories were significantly enriched in HV (Fig. 3a, b), among which four subcategories were potentially involved in environmental adaptation, including ‘response to oxidative stress’ (GO:0006979), ‘response to heat’ (GO:0009408), ‘phagocytosis’ (GO:0006909) and ‘peroxidase activity’ (GO:0004601). In agreement to these findings, a functional enrichment analysis on KEGG pathway showed significant enrichment of ‘endocytosis’ (ko04144), ‘spliceosome’ (ko03040) and ‘metabolism of xenobiotics by cytochrome P450’ (ko00980) in HV (Fig. 3c), but no significantly enriched KEGG pathways were detected in CS. These results indicated that the deep-sea squat lobsters in HV might confront more severe chemical substance threats than those in CS.

The DEGs between HV and CS were associated with diverse gene functions. Specifically, all nine unigenes relevant to antioxidation and detoxification were found to be up-regulated in HV (Table 3), including superoxide dismutase (SOD), peroxidase, dihydrodiol dehydrogenase (DDH), cystathionine gamma-lyase (CSE) and GST. In addition, five DEGs annotated as heat shock proteins (HSPs) were also found to be up-regulated in HV. The up-regulated HSPs included two members of the small HSP family (HSP21 and HSP27) and one member of the HSP70 family (Table 3). In terms of immune-related DEGs, alpha 2-macroglobulin (A2M) was found to be up-regulated in CS, while complement component 1 q (C1q), immunoglobulin (Ig), serine protease and tissue factor pathway inhibitor (TFPI) were expressed consistently more highly in HV than in CS. Meanwhile, five unigenes annotated as CTL were identified as DEGs, of which four were up-regulated in HV. These varied categories of genes with differential expression patterns might be important for *S. crosnieri* to tolerate environmental heterogeneity between HV and CS.

**Table 3:** Summary of DEGs involved in antioxidation and detoxification, heat shock proteins, and immune defense

Gene category	Unigene ID	Expression level		log2(fold change)	*P*adj	Annotation
		HV	CS			
*Antioxidation and detoxification*						
*SOD*	comp117154_c0_seq4	64.14	2.25	5.16	4.11E-10	Superoxide dismutase, Cu-Zn family [*Procambarus clarkii*]
*Peroxidase*	comp78515_c1_seq1	42.17	0.21	8.21	8.24E-08	Peroxidase, putative [*Ixodes scapularis*]
	comp78515_c0_seq1	42.77	0.03	11.10	1.63E-20	Peroxidase-like protein isoform X2 [*Linepithema humile*]
	comp120735_c0_seq1	32.10	1.94	4.16	1.44E-06	peroxidase-like [*Plutella xylostella*]
	comp117925_c0_seq1	320.26	1.31	8.10	5.87E-05	Thyroid peroxidase-like [*Zootermopsis nevadensis*]
*CSE*	comp120565_c0_seq2	24.08	0.10	7.97	1.65E-16	Cystathionine gamma-lyase-like [*Limulus polyphemus*]
*DDH*	comp117984_c0_seq2	114.80	26.70	2.56	1.57E-02	trans-1,2-dihydrobenzene-1,2-diol dehydrogenase-like [*Fopius arisanus*]
*GST*	comp60860_c0_seq1	164.94	41.94	2.13	2.11E-02	Glutathione S-transferases [*Macrobrachium nipponense*]
	comp115940_c0_seq2	1345.57	10.67	7.24	2.57E-07	Glutathione S-transferase s4 [*Lissorhoptrus oryzophilus*]
*Heat shock proteins*						
*HSP*	comp122999_c1_seq6	52.32	5.87	3.39	3.39E-02	Heat shock protein 21 [*Macrobrachium rosenbergii*]
	comp123864_c1_seq1	18.28	2.65	2.90	5.42E-04	Heat shock protein 21 [*Macrobrachium rosenbergii*]
	comp120701_c2_seq3	16.52	2.67	2.86	4.46E-02	Heat shock protein 21 [*Macrobrachium rosenbergii*]
	comp116171_c0_seq1	27.53	4.75	2.76	4.64E-02	Heat shock protein 21 [*Macrobrachium rosenbergii*]
	comp108920_c0_seq1	120.70	27.10	2.43	9.56E-03	Heat shock protein 27, partial [*Procambarus clarkii*]
	comp76811_c0_seq1	36.54	2.64	3.89	9.86E-07	Heat shock protein 70 [*Portunus trituberculatus*]
	comp108897_c0_seq1	18.70	2.44	3.12	1.97E-02	Heat shock protein 70 kDa [*Xantho incisus*]
*Immune defense*						
*CTL*	comp124003_c1_seq3	2124.07	5.61	8.96	4.37E-26	C-type lectin [*Panulirus homarus*]
	comp123320_c0_seq1	201.02	0.90	8.20	9.66E-23	C-type lectin [*Panulirus homarus*]
	comp123413_c2_seq2	2242.87	27.81	6.68	9.16E-15	C-type lectin [*Panulirus homarus*]
	comp107558_c1_seq2	74.66	0.15	9.75	1.23E-17	putative c-type lectin [*Procambarus clarkii*]
	**comp107560_c0_seq1**	**0.09**	**94.83**	**−10.02**	**7.01E-18**	**C type lectin containing domain protein [** ***Macrobrachium nipponense]***
*C1q*	comp123155_c0_seq2	1576.14	3.50	9.21	9.21E-29	putative complement component 1, q subcomponent protein [*Procambarus clarkii*]
	comp117788_c0_seq1	879.58	1.89	9.19	7.82E-13	putative complement component 1, q subcomponent protein [*Procambarus clarkii*]
*TFPI*	comp113374_c0_seq1	210.18	0.45	9.31	2.48E-24	tissue factor pathway inhibitor 2-like [*Cyprinus carpio*]
	comp122322_c0_seq1	665.65	1.61	9.03	8.62E-08	tissue factor pathway inhibitor isoform X4 [*Struthio camelus australis*]
	comp123942_c0_seq1	871.37	2.52	8.82	3.18E-22	Tissue factor pathway inhibitor [*Limulus polyphemus*]
	comp117589_c0_seq1	222.59	0.75	8.64	1.16E-23	Tissue factor pathway inhibitor [*Ophiophagus hannah*]
*Serine protease*	comp121531_c0_seq1	382.44	0.94	9.16	4.37E-26	Serine proteinase-like protein [*Macrobrachium nipponense*]
	comp122830_c0_seq1	10.24	0.07	7.44	3.97E-07	Serine protease 38 precursor [*Nasonia vitripennis*]
	comp118617_c0_seq1	44.91	0.20	7.96	9.48E-20	Serine proteinase stubble-like [*Tropilaelaps mercedesae*]
	comp115439_c0_seq1	8613.91	4331.84	7.09	2.52E-11	putative trypsin serine protease [*Procambarus clarkii*]
*Ig*	comp118268_c0_seq1	47.40	8.50	2.74	2.63E-02	Immunoglobulin [*Oryctes borbonicus*]
*A2M*	**comp101450_c1_seq1**	**0.00**	**14.07**	**-Inf**	**1.77E-02**	**Alpha 2-macroglobulin, partial [** ***Scylla paramamosain]***
	**comp120130_c2_seq1**	**0.00**	**6.95**	**-Inf**	**8.69E-04**	**Alpha-2-macroglobulin [** ***Pacifastacus leniusculus]***

DEGs highlighted in bold are up-regulated in CS

## Discussion

As the typical deep-sea chemosynthetically-driven ecosystems, hydrothermal vents and cold seeps have been reported to differ in many aspects, including geological settings, temperature gradient and chemicals used for energy [3]. Despite their habitat differences, these two types of chemosynthetic ecosystems support dense populations of the same species such as decapod crustaceans. To gather fundamental knowledge about deep-sea adaptation of these specialized macro-benthos to different chemosynthetic environments, we investigated transcriptome profiles of the dominant species, *S. crosnieri* inhabiting both deep-sea vents and seeps. We provided further evolutionary context to our analysis by identifying genes that underwent positive selection. As expected, our analyses identified a number of plausible contributors to the survival of *S. crosnieri* in both deep-sea vents and cold seeps, including genes that act in stress response, immunity defense and genetic information processing. However, these candidates involved in genetic adaptation to different deep-sea chemosynthetic environments in *S. crosnieri* should be verified in the future.

### Signals of positive selection in *S. crosnieri* during adaptation into deep-sea vents and seeps

Transcriptome-wide analysis of the rates of non-synonymous to synonymous nucleotide substitutions represents a promising approach to quantitatively measure the selection force by identifying genes under positive selection [44, 45]. The heterogeneous environmental conditions between hydrothermal vents and cold seeps can be an important selective force driving adaptive divergence of macro-organisms living in both habitats. In this study, we used the maximum likelihood approach to trace signals of positive selection in *S. crosnieri* during adaptation into deep-sea vents and seeps and detect positively selected sites of functional genes (Additional file 7: Figure S4). A total of 82 genes were identified to be positively selected and involved in many biological processes, including antioxidation, detoxification, immunity and genetic information processing.

**Fig. 4:**
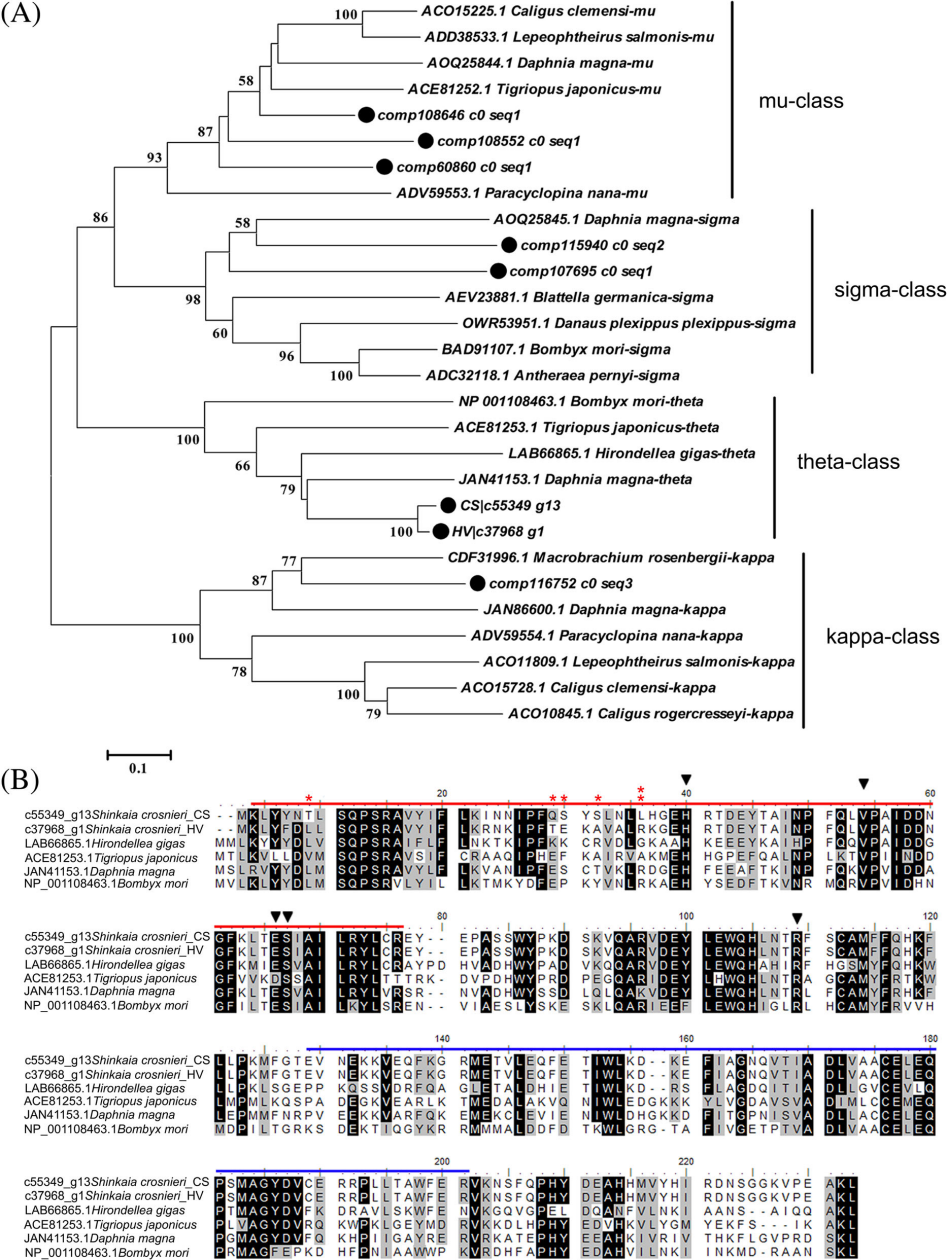
Sequence analysis of the glutathione S-transferase (GST) from *S. crosnieri*. **a** Phylogenetic analysis of amino acid sequences of GSTs from *S. crosnieri* (marked with circles) and those from arthropods using the maximum likelihood method. In the phylogenetic tree, each entry contains the accession number, species name and GST class. Bootstrap values (> 50%) are indicated at branch nodes. **b** Alignment of amino acid sequences of theta-class GSTs in *S. crosnieri* with other four arthropods. The N-terminal domain is indicated by the red line; the C-terminal domain is indicated by the blue line; the putative GSH-binding sites (G-sites) are represented by triangles. Double asterisks stand for the amino acids in *S. crosnieri* with a BEB posterior probability that is larger than 0.99, and one asterisk stands for the sites with a posterior probability higher than 0.95 but lower than 0.99

Genes that related to stress response have been reported to evolve under positive selection in diverse groups of organisms, including algae [46], insects [47, 48] and mussels [10]. Representative genes involved in antioxidation and detoxification were also found to be positively selected in *S. crosnieri* between HV and CS. Enzymes encoded by these genes include Trxs which act as antioxidants by catalyzing the reduction of other proteins by cysteine thiol-disulfide exchange [49], rhodanese that functions to detoxify cyanide into relatively nontoxic thiocyanate [50], and GSTs involved in the cellular detoxification of various endogenous and exogenous electrophilic compounds [51]. As in GST, four putative GST classes (mu, sigma, theta, and kappa) were identified in *S. crosnieri* transcriptomes based on phylogenetic analysis of GSTs among different arthropod species (Fig. 4a). Positively selected mutations were only detected in the gene encoding theta-class GST in *S. crosnieri* (ScGSTT), which have resulted in 13 amino acid substitutions (Fig. 4b). Homologous sequence comparison revealed that ScGSTT included the N-terminal and C-terminal domains, glutathione binding sites, as well as other conserved residues of theta-class GSTs (Fig. 4b) [52]. It is noteworthy that almost all positively selected sites are located in the vicinity of the active site of the N-terminal domain in ScGSTT. It has been shown that the amino acid diversity at the N- and C- terminal binding domains of GST defines substrate selectivity [52, 53]. We speculate that divergent evolution of key functional domains of ScGSTT has probably contributed to different stress tolerance capabilities of *S. crosnieri* to survive in different deep-sea chemosynthetic environments. However, the specific feature and function of ScGSTT should be pursued experimentally in the future.

**Fig. 5:**
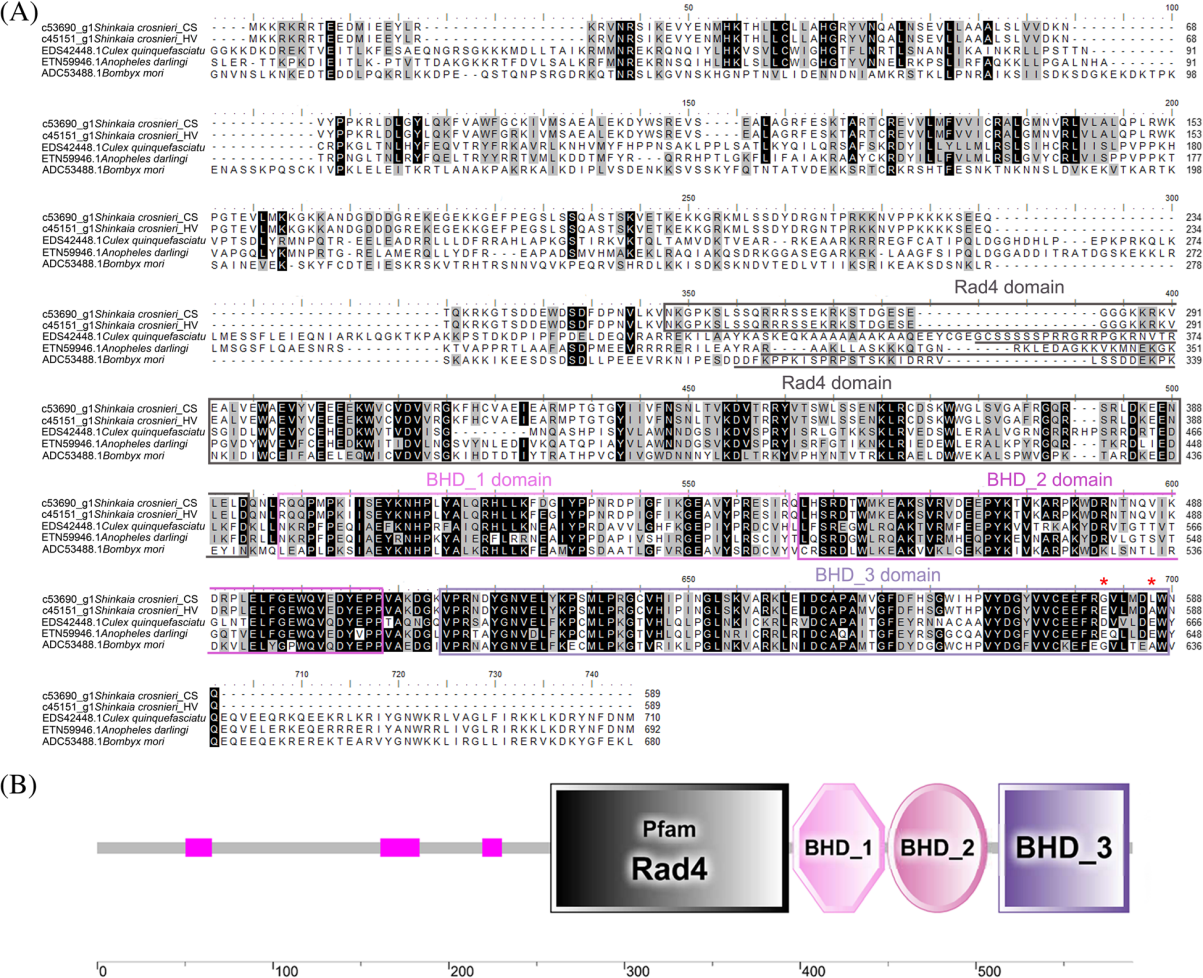
Sequence analysis of DNA repair protein RAD4 identified in *S. crosnieri*. **a** The alignment of deduced RAD4 amino acid sequences in *S. crosnieri* with other three arthropods. The conserved functional domains of RAD4 were highlighted in lines or in boxes. The asterisk stands for the amino acids in *S. crosnieri* with a posterior probability higher than 0.95 but lower than 0.99. **b** Functional domains in RAD4 from *S. crosnieri*. Pfam Rad4: Rad4 transglutaminase-like domain; BHD: beta-hairpin domain. The pink squares represent low complexity regions

In addition to stress response genes, immune-related genes, such as CTLs which are responsible for the initiation of the host immune response against pathogen [54], and AMPs which are important effector protein of innate immunity [55], were also found to harbor positively selected mutations (Additional file 7: Figure S4A). Positive selection in genes related to immune response might be a consequence of adaptive evolution in *S. crosnieri* during adaptation into deep-sea vents and seeps when confronted with different environmental stressors, such as diverse pathogens. We also noticed that a couple of genes involved in DNA repair and translation regulation have evolved under positive selection (Additional file 7: Figure S4B). For example, RAD4, known as a repair factor, plays a key role in the detection of DNA damage and initiation of DNA repair [56]. The serine/arginine-rich splicing factors participate in a wide range of alternative splicing events and can function as modulator of DNA repair [57]. The p53 protein is widely involved in numerous cellular processes, including DNA transcription, DNA repair, apoptosis as well as maintenance of genomic stability [58]. In the case of RAD4, sequence analysis showed that RAD4 protein identified from *S. crosnieri* transcriptomes contained a typical transglutaminase-like domain and three beta-hairpin domains (BHDs) that are responsible for DNA binding (Fig. 5). Previous biochemical and cellular studies have shown that beta-hairpin from BHD_3 plays an import role in the recognition of DNA damage [59, 60]. Substitution analysis of RAD4 in the vent and seep squat lobster by the site model showed that BHD_3 was positively selected (Fig. 5a), indicating different environmental conditions could have affected the ability of RAD4 to recognize DNA damage. Ample evidences indicated the effect of environmental exposure to heavy metals and heat stress on the integrity of DNA and the repair system [61, 62]. Hydrothermal vents, usually characterized with high temperature, sulphide and heavy metal concentrations, seem more severe than seeps as environments for organisms [3]. Therefore, the positive selection in those key genes associated with DNA repair may imply their roles for *S. crosnieri* to prevent potential heat stress- and heavy metal-induced DNA damage in hydrothermal vents.

### DEGs involved in adaptation to deep-sea vents and seeps in *S. crosnieri*

It has been known that environmental stresses can lead to increased cellular levels of reactive oxygen species (ROS), which can pose a threat to cells by causing oxidative damage on a wide diversity of molecules, especially proteins and lipids [63, 64]. Aerobic organisms can adapt to the enhanced ROS production during environmental stresses by up-regulating antioxidant defenses [65]. Compared to cold seeps, hydrothermal vents seem more severe as environments for organisms [3]. Concordantly, our study showed that a number of antioxidant genes were up-regulated in HV. Proteins encoded by these genes either contributed to directly scavenge ROS (e.g., SOD and peroxidase), or participated in protecting cells from toxic effects of ROS and repairing damaged cellular components targeted by ROS (e.g., GSTs and HSPs). Among up-regulated genes in HV, we detected one unigene annotated as copper/zinc superoxide dismutase (Cu/Zn SOD), which catalyzes the dismutation of superoxide into molecular oxygen and hydrogen peroxide (H_2_O_2_), and four unigenes annotated as peroxidase, which catalyzes the reduction of H_2_O_2_ into water and oxygen. Cu/Zn SOD is a copper- and zinc-containing superoxide dismutase that is crucial in preventing cells from oxidative damage [66]. Moreover, Cu/Zn SOD has also been reported to be involved in detoxification and innate immune response of crustaceans [67, 68]. Furthermore, two genes coding for proteins related to CSE and DDH were also up-regulated in HV. Recent studies have demonstrated the antioxidative property of CSE and suggested that CSE is a critical factor in regulating the synthesis of glutathione, the first-line antioxidative defense [69, 70]. DDH belongs to a family of aldo-keto reductases that are involved in detoxification of environmental mutagenic hazards [71]. Besides detoxification, this enzyme could be involved in regulation of intercellular ROS levels and its induction may act as a response to oxidative stress [72, 73]. Up-regulation of these gene expression might render *S. crosnieri* individuals in HV with a stronger antioxidant defense against ROS.

Increasing evidence has revealed that ROS can cause peroxidation of lipids, resulting in the formation of electrophilic substrates toxic to living organisms [74, 75]. The defense mechanisms against lipid peroxidation constitute the second line of defense against oxidative stress. In this study, two unigenes encoding enzymes related to glutathione-dependent detoxification, GSTs, were found to be up-regulated in HV. Besides the protective effects against xenobiotics, GSTs also play a crucial role in protecting organisms from lipid peroxidation via detoxification of lipid peroxidation end products [75, 76]. In addition to up-regulating genes related to direct elimination of ROS or detoxification of electrophilic products during oxidation process, *S. crosnieri* also up-regulated gene expression of HSPs in HV against stress-activated protein misfolding and aggregation. It is well known that exposure of cells to environmental stresses can induce gene expression of HSPs that function as molecular chaperones to repair or degrade damaged proteins as cellular defenses [77]. Taken together, these results indicated that *S. crosnieri* might up-regulate a battery of genes associated with stress response to increase its capability to manage more environmental stresses in the hydrothermal vents.

Similarly, a number of immune-related genes were found to be differentially expressed, including two types of pattern recognition receptors (PRRs) that can recognize conserved molecular structures on pathogens called pathogen-associated molecular patterns (PAMPs) [78]. Of all the PRRs families, CTLs play a critical role in initiating and regulating the host immune response against pathogen exposure, which have been demonstrated to function importantly in anti-microbial immunity of many crustaceans [54, 79, 80]. Another type of differentially expressed PRRs was a C1q homolog, which contains a conserved C1q domain (Additional file 8: Figure S5). Known as a versatile PRR in innate immunity, C1q can recognize and bind to various PAMPs, and then trigger the classical complement pathway [81]. In addition to this, it is established that C1q is involved in clearance of apoptotic cells upon phagocytosis, thereby playing a major role in maintenance of immune tolerance [82, 83]. Interestingly, most differentially expressed lectin and C1q were significantly up-regulated in HV, indicating microbial communities encountered by *S. crosnieri* might differ between HV and CS.

Besides PRRs, genes coding for enzymes/proteins involved in the downstream immune responses were also identified in DEGs. Among them, TFPI, serine protease and Ig were consistently up-regulated in HV. As a major regulator of blood clotting, TFPI has also been shown to possess anti-microbial properties that can execute pathogen killing [84]. Serine protease has been shown to participate in diverse defense responses in invertebrates, including synthesis of antimicrobial peptide, hemolymph coagulation and melanotic encapsulation [85]. Igs are the key components of adaptive immune system by specifically recognizing and neutralizing invading pathogens, such as bacteria and viruses [86]. Although adaptive immunity has long been considered to be an evolutionary innovation unique to vertebrates, Ig-mediated adaptive immunity has been recently reported to be involved in host defense of several crustaceans, including water fleas *Daphnia* [87], the white shrimp *Litopenaeus vannamei* [88] and the crayfsh *Procambarus clarkii* [89]. The gene coding for Ig in *S. crosnieri* harbored one Ig-like domain (Immunoglobulin C-2 Type, IGc2) (Additional file 9: Figure S6), implying its alternative adaptive immunity. However, further experiments are needed to determine the exact function of Ig in *S. crosnieri*. On the other side, two unigenes coding for A2M showed higher expression in CS. As a broad-spectrum proteinase, A2M functions in delivering proteinase to an endocytotic proteinase clearance pathway, thereby protecting organisms against invading pathogens [90], which has been reported in shrimp [91]. Immune gene expression differences between HV and CS suggested that *S. crosnieri* possibly performed specific immune response that generated diverse pathogen resistance to support individual adaptation when confronting different environmental stressors. Together with our finding that stress response and immune-related genes are also positively selected in *S. crosnieri* between HV and CS, overexpression of a battery of antioxidant and immune genes in HV suggests that oxidative stress and immune defense are more active in vent *S. crosnieri*. It might be a consequence of more environmental stresses encountered by *S. crosnieri* in hydrothermal vents than cold seeps.

## Conclusions

This study represented a first step in understanding the deep-sea adaptation mechanism of the galatheid squat lobster *S. crosnieri* at the molecular level, and provided a comprehensive transcriptomic resource for molecular studies of this species. Through the maximum likelihood approach, we identified traces of positive selection and detected amino acid substitutions in specific proteins, which revealed that adaptation was driving the evolution of key genes associated with adaptation to deep-sea vents and seeps in *S. crosnieri*. In addition, a large number of DEGs were detected between HV and CS, and most of the DEGs associated with stress response and immunity were up-regulated in HV, suggesting that hydrothermal vents might be more severe as environments for *S. crosnieri* in comparison with cold seeps. Moreover, a number of stress response and immune-related genes were found positively selected and/or differentially expressed, highlighting the importance of those key genes in adaptation of *S. crosnieri* to deep-sea vents and seeps. Overall, our results indicated that the capacity of *S. crosnieri* to thrive in different deep-sea chemosynthetic environments derived partly from adaptive evolution of functional genes associated with stress response and immunity, and alterations in their gene expression that lead to different stress resistance. Since transcriptomic analysis has limitations to capture signals of positive selection occurring in the regulatory regions, and mRNA levels are not necessarily correlated with protein abundance or activity due to post-translation modifications, further studies including systematic genomic and proteomic analyses are required to validate the results and test the proposed hypotheses.

## Additional files

Additional file 1: **Table S1.** Gene expression analyzed by qRT-PCR and RNA-Seq. (XLSX 11 kb)

Additional file 2: **Figure S1.** GO classification (A), KOG function classification (B), and KEGG pathway classification (C) of all unigenes in the transcriptome of *Shinkaia crosnieri*. (PDF 16847 kb)

Additional file 3: **Figure S2.** Phylogenetic tree of three arthropods based on the orthologous genes. (PDF 44 kb)

Additional file 4: **Table S2.** The list of positively selected genes between vent and seep *Shinkaia crosnieri*. (XLSX 24 kb)

Additional file 5: **Table S3.** Differential expressed unigenes between vent and seep *Shinkaia crosnieri*. (XLSX 59 kb)

Additional file 6: **Figure S3.** Heatmap of all DEGs. (PDF 14963 kb)

Additional file 7: **Figure S4.** Partial alignment of positively selected genes. (A) Positively selected genes related to stress response and immunity. (B) Positively selected genes related to genetic information progressing. (PDF 141 kb)

Additional file 8: **Figure S5.** Deduced amino acid sequence alignment of comp123155_c0_seq2, comp117788_c0_seq1 with complement component 1 q (C1q) from *Procambarus clarkia* (ASC55672), *Lingula anatine* (XP_013406649), and *Crassostrea virginica* (XP_022323368). Conserved amino acid residues were in colors. The highly conserved C1q domain was highlighted in a black box. (PDF 228 kb)

Additional file 9: **Figure S6.** Deduced amino acid sequence of immunoglobulin (Ig) in *Shinkaia crosnieri*. The Ig-like domain (Immunoglobulin C-2 Type, IGc2) was highlighted in a black box. The internal repeats were underlined. (PDF 276 kb)

## Abbreviations

A2M: Alpha 2-macroglobulin
AMP: Antimicrobial peptide
ANOVA: One-way analysis of variance
BEB: Bayes Empirical Bayes
C1q: Complement component 1 q
CDS: Coding sequences
CS: A cold seep in the South China Sea
CSE: Cystathionine gamma-lyase
CTL: C-type lectin
Cu/Zn SOD: Copper/zinc superoxide dismutase
DDH: Dihydrodiol dehydrogenase
DEG: Differentially expressed gene
d_N_: Nonsynonymous nucleotide substitutions per site
d_S_: Synonymous nucleotide substitutions per site
FDR: False discovery rate
FPKM: Fragments per kilobase of exon model per million mapped reads
GO: Gene Ontology
GST: Glutathione S-transferase
H_2_O_2_: Hydrogen peroxide
HSP: Heat shock protein
HSPG: Heparan sulfate proteoglycans
HV: The Iheya North hydrothermal vent
Ig: Immunoglobulin
IGc2: Immunoglobulin C-2 Type
KEGG: Kyoto Encyclopedia of Genes and Genomes
KOG: euKaryotic Ortholog Group
LRT: Likelihood ratio test
Nr: Non-redundant protein database
PAMP: Pathogen-associated molecular pattern
Pfam: Protein Family
PP: Posterior probability
PRR: Pattern recognition receptor
PSG: Positively selected gene
qRT-PCR: Quantitative real-time PCR
ROS: Reactive oxygen species
ROV: Remotely operated vehicle
SOD: Superoxide dismutase
SRA: Sequence Read Archive
TFPI: Tissue factor pathway inhibitor
Trx: Thioredoxin
