# Supplementary material for: Transcriptomic analysis reveals insights into deep-sea adaptations of the dominant species, Shinkaia crosnieri (Crustacea: Decapoda: Anomura), inhabiting both hydrothermal vents and cold seeps
Source: BMC Genomics. 2019 May 18;20:388. doi: 10.1186/s12864-019-5753-7 (PMC6525460; doi:10.1186/s12864-019-5753-7)
Supplement: Supplementary file 2 — Figure S1. GO classification (A), KOG function classification (B), and KEGG pathway classification (C) of all unigenes in the transcriptome of Shinkaia crosnieri. (PDF 16847 kb) [file 12864_2019_5753_MOESM2_ESM.pdf]

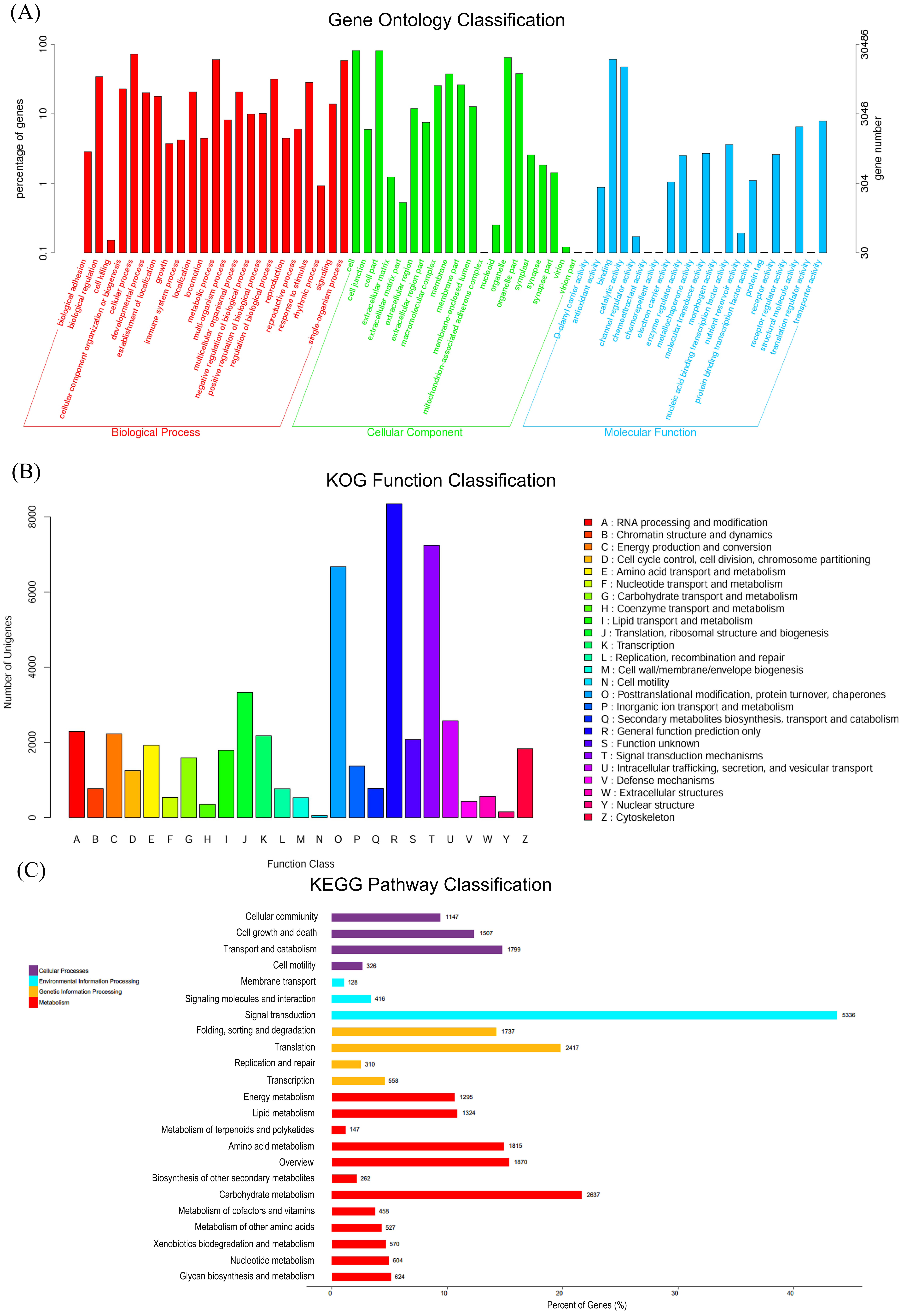

Additional file 2: Figure S1 GO classification (A), KOG function classification (B), and KEGG pathway classification (C) of all unigenes in the transcriptome of *Shinkaia crosnieri*.
