# Supplementary material for: Transcriptomic analysis reveals insights into deep-sea adaptations of the dominant species, Shinkaia crosnieri (Crustacea: Decapoda: Anomura), inhabiting both hydrothermal vents and cold seeps
Source: BMC Genomics. 2019 May 18;20:388. doi: 10.1186/s12864-019-5753-7 (PMC6525460; doi:10.1186/s12864-019-5753-7)
Supplement: Supplementary file 3 — Figure S2. Phylogenetic tree of three arthropods based on the orthologous genes. (PDF 44 kb) [file 12864_2019_5753_MOESM3_ESM.pdf]

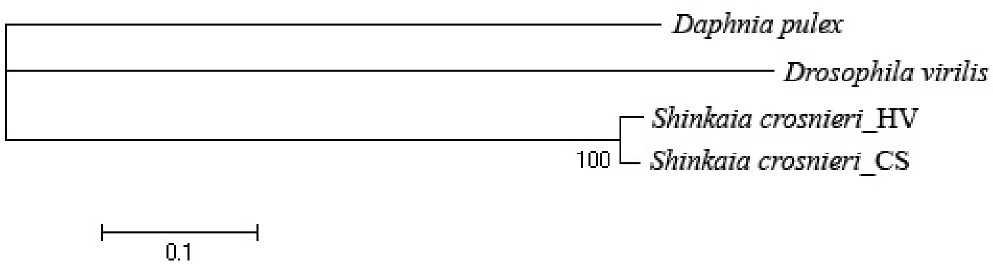

Additional file 3: Figure S2 Phylogenetic tree of three arthropods based on the orthologous genes.
