# Supplementary figures and images for: Transcriptomic analysis reveals insights into deep-sea adaptations of the dominant species, Shinkaia crosnieri (Crustacea: Decapoda: Anomura), inhabiting both hydrothermal vents and cold seeps
Source: BMC Genomics. 2019 May 18;20:388. doi: 10.1186/s12864-019-5753-7 (PMC6525460; doi:10.1186/s12864-019-5753-7)

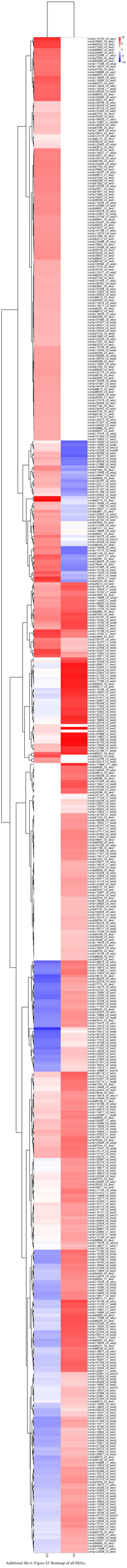

Additional file 6: Figure S3 Heatmap of all DEGs.

Supplement: Supplementary file 6 — Figure S3. Heatmap of all DEGs. (PDF 14963 kb) [file 12864_2019_5753_MOESM6_ESM.pdf]
