# Supplementary material for: Transcriptomic analysis reveals insights into deep-sea adaptations of the dominant species, Shinkaia crosnieri (Crustacea: Decapoda: Anomura), inhabiting both hydrothermal vents and cold seeps
Source: BMC Genomics. 2019 May 18;20:388. doi: 10.1186/s12864-019-5753-7 (PMC6525460; doi:10.1186/s12864-019-5753-7)
Supplement: Supplementary file 7 — Figure S4. Partial alignment of positively selected genes. (A) Positively selected genes related to stress response and immunity. (B) Positively selected genes related to genetic information progressing. (PDF 141 kb) [file 12864_2019_5753_MOESM7_ESM.pdf]

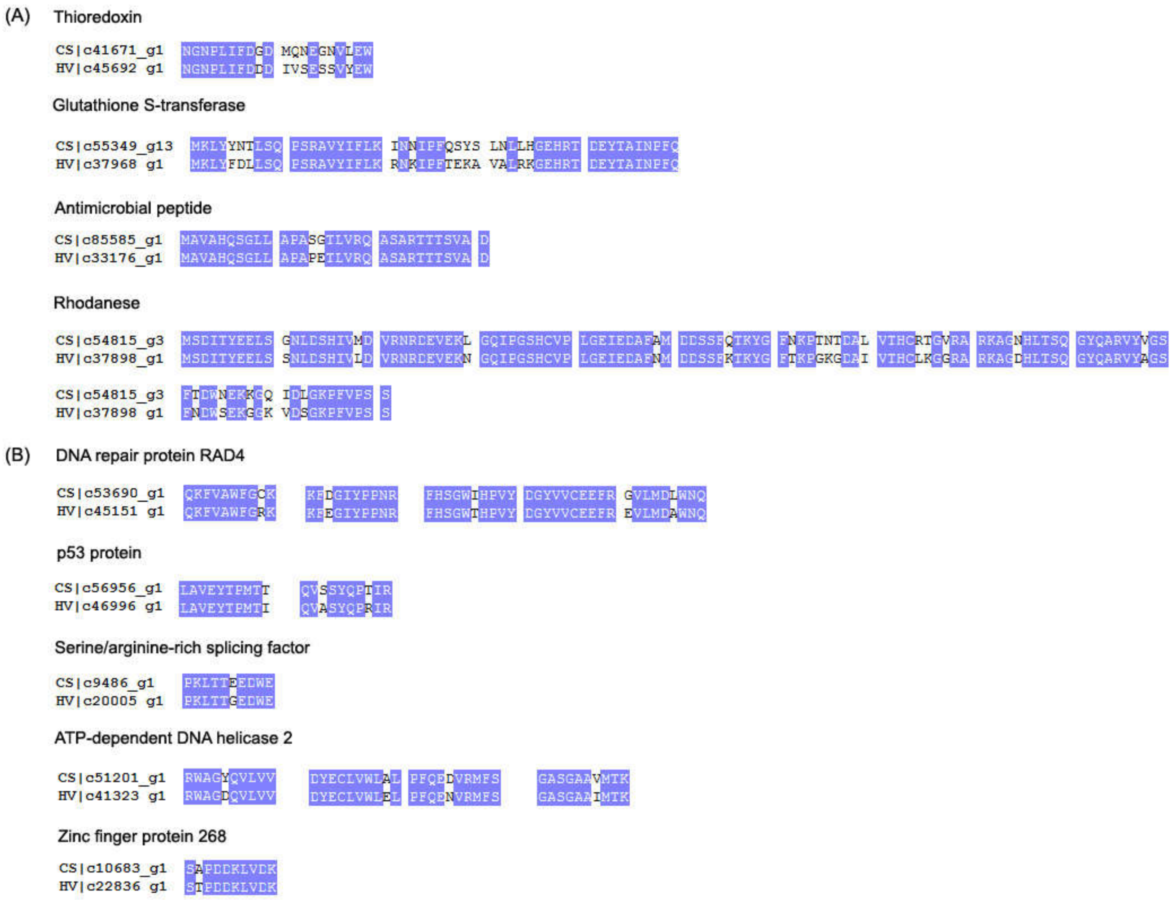

Additional file 7: Figure S4 Partial alignment of positively selected genes. (A) Positively selected genes related to stress response and immunity. (B) Positively selected genes related to genetic information progressing.
