# Supplementary material for: Transcriptomic analysis reveals insights into deep-sea adaptations of the dominant species, Shinkaia crosnieri (Crustacea: Decapoda: Anomura), inhabiting both hydrothermal vents and cold seeps
Source: BMC Genomics. 2019 May 18;20:388. doi: 10.1186/s12864-019-5753-7 (PMC6525460; doi:10.1186/s12864-019-5753-7)
Supplement: Supplementary file 8 — Figure S5. Deduced amino acid sequence alignment of comp123155_c0_seq2, comp117788_c0_seq1 with complement component 1 q (C1q) from Procambarus clarkia (ASC55672), Lingula anatine (XP_013406649), and Crassostrea virginica (XP_022323368). Conserved amino acid residues were in colors. The highly conserved C1q domain was highlighted in a black box. (PDF 228 kb) [file 12864_2019_5753_MOESM8_ESM.pdf]

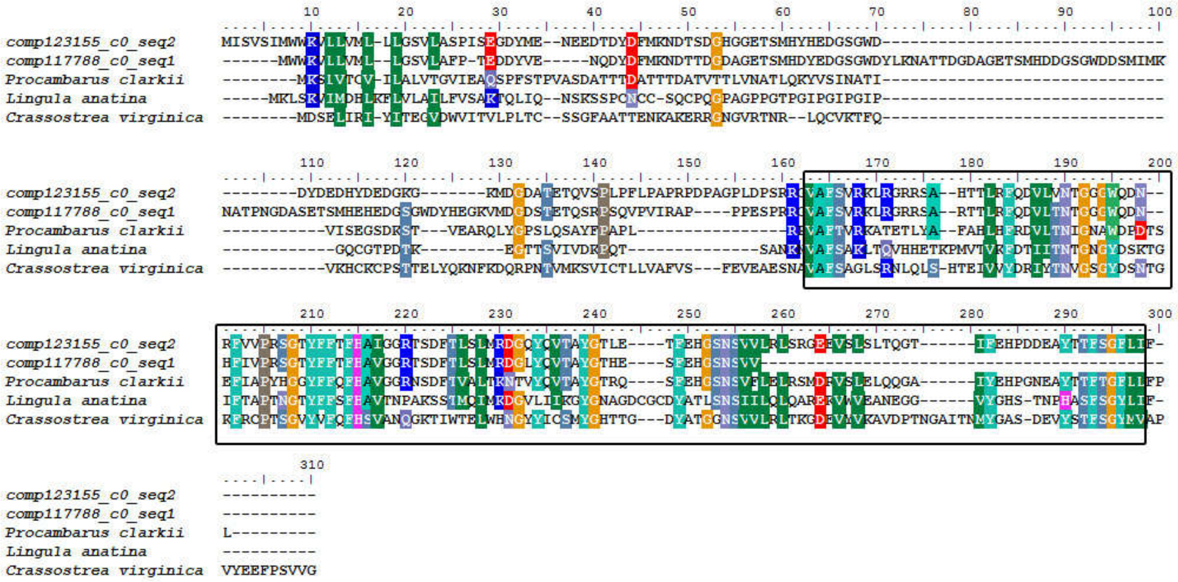

Additional file 8: Figure S5 Deduced amino acid sequence alignment of comp123155_c0_seq2, comp117788_c0_seq1 with complement component 1 q (C1q) from *Procambarus clarkia* (ASC55672), *Lingula anatine* (XP_013406649), and *Crassostrea virginica* (XP_022323368). Conserved amino acid residues were in colors. The highly conserved C1q domain was highlighted in a black box.
