# Supplementary material for: Transcriptomic analysis reveals insights into deep-sea adaptations of the dominant species, Shinkaia crosnieri (Crustacea: Decapoda: Anomura), inhabiting both hydrothermal vents and cold seeps
Source: BMC Genomics. 2019 May 18;20:388. doi: 10.1186/s12864-019-5753-7 (PMC6525460; doi:10.1186/s12864-019-5753-7)
Supplement: Supplementary file 9 — Figure S6. Deduced amino acid sequence of immunoglobulin (Ig) in Shinkaia crosnieri. The Ig-like domain (Immunoglobulin C-2 Type, IGc2) was highlighted in a black box. The internal repeats were underlined. (PDF 276 kb) [file 12864_2019_5753_MOESM9_ESM.pdf]

```
        10         20         30         40         50         60         70         80         90        100
MKDESDVSTD SGRTQKKVEF HTETHVEVMS DKKVEVFQSS SVDVSPKPTK KPGFKQEHKF ERKVIVEPKI EKPHQVSKTF QPLKETPKPS SHEIRYEKSK

       110        120        130        140        150        160        170        180        190        200
VTSEMSEKKE HTAKTVVFKK TVETSKVDGV KKVQQPSEPR MIKPTIAQPS VPHVEPLKST GLVKQAISKQ ELQQELDLKP FPFKAEPSKP KKPRGVPPGQ

       210        220        230        240        250        260        270        280        290        300
PTKFMKGEFH ESDYDSDYEG RIPPKWKPAD SDTEDQNYGS VKAPSGTYKA PEPHRRTPTP PTAFDASPKY DGRPRPKIDF PESEPEPERD VSPEIVIPEK
                          Internal repeat 1
       310        320        330        340        350        360        370        380        390        400
VEVVKEPVPP KIVPKEAKIF KSQPQKPKPP RVRVPSPELK PGSPPIEDYA PPPKRPPPPP QPSPFSNAVG VESTKITKIA DSSSHHQRFV TMQQTTRVIK

       410        420        430        440        450        460        470        480        490        500
FTDGRSSSTR TETQETQAHE PRGRKVVREE QKPLPPLEPF PFSPDPAKPR KERGGPPTKP RKFRKGEFTE SDYESDYEGP IKPKWQPPDS DTDDPSYKKV

       510        520        530        540        550        560        570        580        590        600
KTDIRYDRTP SKTRDRTPTP PTVFDTPPES GGPWRPVIKP EPVLQREPSP EVLVPREPPK SAHVVKKIAH KVQQEVVKLV ERAASPPLPP PGTPPEEGVI

       610        620        630        640        650        660        670        680        690        700
LQETQYVVDR VDLQLKVKAP PMKEVQTYED KPYTKVTERT EKTFTNIKLQ EELHMKTEKE ETRKTIVTEY EQVPLHPLPS LKTEVTVSME KIQDLEPFPF

       710        720        730        740        750        760        770        780        790        800
EPGKGKPKRE RGPPPPKPKK FVKGEFQESD YDSDFEGRVR PKWQPASSDV EDPEYTPVKP PPTLGKQTYG SKNRRSPTPP TKFEVPESSG GPLRPNIEPA
                                                  Internal repeat 1
       810        820        830        840        850        860        870        880        890        900
EIPVKIIREP SPEIIIPKVK EKLVTKALKV TPKAPALKVG EPAAPRPRTP TPPLPEPGPQ PEIGYIPGKV MHREEEDVHI RMIKKKIEAK KGFQIDIDVT

       910        920        930        940        950        960        970        980        990       1000
DIYDFVSESE YEKTDVEANK MIKPFPPLEP FPYEPDPSRP KRQRGPPPPH PKKFMKGEFR GSDYESDYDR PIAPKWVPPD SEGEEITYRR VAPPVVDSVR

      1010       1020       1030       1040       1050       1060       1070       1080       1090       1100
HRSESSGRDP SPPSKFDQPP HFEGPPRPVL DPSDLPRRER RESLEEYSIP RFPKVEFKPF DLEDEQVSGP QVAAVTTDTE TEPESYAQGG IDRKYVKSAQ

      1110       1120       1130       1140       1150       1160       1170       1180       1190       1200
KVVGHQFEDM TQTFRHKAQR FAEQLVTEVF AAREGSEPAE PAGEPPSLPE ELKAQDDSTE AATEPPPPPD LAAQKALSPS YQEPQAYRDE SRISEFGTKH

      1210       1220       1230       1240       1250       1260       1270       1280       1290       1300
IDPDTGLIYF KYDFGYEFGV ILPGEAKKVE KKREMNGDHS TDIPIPVIHE PTASSQKPDK KIPKSNGHVP HLEGGHPKHP SGAVTQSLAP TIGPSAQFPG

      1310       1320       1330       1340       1350       1360       1370       1380       1390       1400
TEQVSPHPSH QSDISETDRE YQQYMGKMIP EFVPKKQAQF RPISGDPYSD SEAESDYPSP SKLFDVGPGV HGGPKRFMPV VPGAAVQPKS HPPQGPLPEA

      1410       1420       1430       1440       1450       1460       1470       1480       1490       1500
SCTTAELGVL GSPLSATPPS TPSTPCSGPL TQTTRPLHYI TPLRDLSVAS GEALKLECVV QADPSVQVSW SQGGDVLYPS PHYQMVYRNG VCRLIIPCVS
                                                                                IGc2 domain
      1510       1520       1530
PDDEGVYTCT AVGVTGMDST SATVCITGEK K
```

Additional file 9: Figure S6 Deduced amino acid sequence of immunoglobulin (Ig) in *Shinkaia crosnieri*. The Ig-like domain (Immunoglobulin C-2 Type, IGc2) was highlighted in a black box. The internal repeats were underlined.
